# Susceptibility to hypertension based on *MTHFR* rs1801133 single nucleotide polymorphism and *MTHFR* promoter methylation

**DOI:** 10.3389/fcvm.2023.1159764

**Published:** 2023-10-02

**Authors:** Ming-Huang Chiu, Chia-Hsiu Chang, Disline Manli Tantoh, Tsui-Wen Hsu, Chih-Hsuan Hsiao, Ji-Han Zhong, Yung-Po Liaw

**Affiliations:** ^1^ Department of Pulmonology and Respiratory Care, Cathay General Hospital, Taipei City, Taiwan; ^2^ Cardiovascular Center, Cathay General Hospital, Taipei City, Taiwan; ^3^ Department of Medical Imaging, Chung Shan Medical University Hospital, Taichung City, Taiwan; ^4^ Department of Public Health and Institute of Public Health, Chung Shan Medical University, Taichung City, Taiwan; ^5^ Superintendent Office, Institute of Medicine, Cathay General Hospital, Taipei City, Taiwan; ^6^ Institute of Medicine, Chung Shan Medical University, Taichung City, Taiwan

**Keywords:** *MTHFR*, promoter methylation, rs1801133, hypertension, susceptibility, interaction

## Abstract

**Background:**

The aetio-pathologenesis of hypertension is multifactorial, encompassing genetic, epigenetic, and environmental factors. The combined effect of genetic and epigenetic changes on hypertension is not known. We evaluated the independent and interactive association of *MTHFR* rs1801133 single nucleotide polymorphism (SNP) and *MTHFR* promoter methylation with hypertension among Taiwanese adults.

**Methods:**

We retrieved data including, *MTHFR* promoter methylation, *MTHFR* rs1801133 genotypes (CC, CT, and TT), basic demography, personal lifestyle habits, and disease history of 1,238 individuals from the Taiwan Biobank (TWB).

**Results:**

The distributions of hypertension and *MTHFR* promoter methylation quartiles (β < 0.1338, 0.1338 ≤ β < 0.1385, 0.1385 ≤ β < 0.1423, and β ≥ 0.1423 corresponding to <Q1, Q1–Q2, Q2–Q3, and ≥Q3) among individuals with the rs1801133 genotypes (CC, CT, and TT) were significantly different (*P* < 0.05). The risk of hypertension was significantly higher among individuals with the TT genotype compared to the reference genotype (CC): odds ratio (OR); 95% confidence interval (CI) = 2.718; 1.503–4.914. The trend of the association of the CT and TT genotypes with hypertension was dose-dependent (*P-trend* = 0.0041). *MTHFR* promoter methylation (lower quartiles compared to ≥Q3) was not significantly associated with hypertension. However, its interaction with *MTHFR* rs1801133 was significant (*P* = 0.0323). After stratification by rs1801133 genotypes, lower *MTHFR* promoter methylation quartiles (<Q1, Q1–Q2, Q2–Q3) compared to ≥Q3 were significantly associated with a higher risk of hypertension among individuals carrying the CC genotype: ORs (95% CIs) = 3.225 (1.140–9.124), 4.177 (1.424–12.247), and 8.645 (2.513–29.739) for Q2–Q3, Q1–Q2, and <Q1, respectively. The trend test was significant (*P-trend* = 0.0009).

**Conclusion:**

Independently, rs1801133 TT was associated with a higher risk of hypertension, but methylation was not. Based on genotypes, lower methylation was dose-dependently associated with a higher risk of hypertension in individuals with the CC genotype. Our findings suggest that *MTHFR* rs1801133 and *MTHFR* promoter methylation could jointly influence hypertension susceptibility.

## Introduction

A single nucleotide polymorphism (SNP) is a genetic change at a specific location in a DNA sequence, where a single nucleotide (A, T, C, or G) is replaced by another in at least 1% of the population (1). Epigenetic changes are modifications in gene expression due to physiological or environmental stimuli (2, 3). DNA methylation is a heritable epigenetic regulatory mechanism that affects gene transcription and expression (4–8). Promoter DNA methylation is the addition of a methyl group to the C-5 position of a cytosine in the promoter region, forming 5-methylcytosine (7).

Hypertension is a life-threatening non-communicable disease characterized by abnormally higher blood pressure. Hypertension is the leading cause of premature death worldwide (9). Globally, approximately 1.278 billion adults aged between 30 and 79 were hypertensive in 2019 (10). The disease could affect about 1.56 billion adults worldwide in 2025 (11). Hypertensive patients do not present with typical symptoms, prompting the need for early identification and regular follow-up of high-risk individuals for effective implementation of preventive and therapeutic management measures (12, 13).

Hypertension has a multifaceted pathophysiology, originating from genetic, epigenetic, and environmental sources, alongside a complex interplay of factors (2, 14–19). So far, only about 10% of the hypertension etiology is known (20). Some known risk determinants of hypertension include SNP, DNA methylation, alcohol drinking, cigarette smoking, inactive lifestyle, sex, body mass index (BMI), and age (20–27). Genome-wide association studies —GWAS— (28–31) and subsequent studies including meta-analyses (26, 27, 32–34) have identified several hypertension-related single nucleotide polymorphisms (SNPs). The known loci explain only about 41% of hypertension heritability (35). Since DNA methylation also affects hypertension (2), gene-environment interactions could account for the remaining heritability (3). Moreover, since the genome and epigenome interwind (36), combining the genetic and epigenetic biomarkers could improve risk stratification and identify potential targets for pharmacological and lifestyle interventions (32, 37, 38).

*MTHFR* is a regulatory enzyme that plays a pivotal role in folate and homocysteine metabolism by catalyzing the synthesis of the main circulatory folate (5methylenetetrahydrofolate) from 5 to 10-methylenetetrahydrofolate (39). This *MTHFR*-catalyzed folate metabolism pathway plays a role in DNA methylation through methylenetetrahydrofolate, which acts as a co-substrate for the methylation of homocysteine to methionine, generating the methyl donor, S-adenosylmethionine (39, 40). Hypermethylation of *MTHFR* and the resulting decrease in gene expression is associated with several conditions and disorders, including but not limited to oxidative stress, diabetes, diabetic complications, ischemic stroke, and cancer (41–43).

A1298C (rs1801131) and C677T (rs1801133) are the major *MTHFR* variants that impair the gene’s activities (44, 45). A1298C results from adenine (A) to cytosine (C) substitution at position 1,298 in exon 7 of *MTHFR* while C677T (rs1801133) results when thymidine (T) replaces cytosine (C) at position 677 (45). C677T is the most common and broadly studied *MTHFR* variant (46). Even though several studies found *MTHFR* rs1801133 as an independent risk promoter for hypertension in different populations (10, 19, 47–52), a study among Mexicans found a significant protective effect of the variant on hypertension (21). Other studies involving Danish (53), Chinese (54), Caucasians (55), and Japanese (56) found no significant association between rs1801133 and hypertension. To our knowledge, only one study (with only 173 participants) assessed the association between rs1801133 and hypertension among Taiwanese (48). Moreover, no study determined the risk of hypertension in relation to both *MTHFR* methylation and SNP among Taiwanese.

The inconsistent results and inadequate studies in some populations pave the way for further investigations (50). Moreover, despite being the most common and broadly studied variant, the mechanistic insights into the causative role of *MTHFR* rs1801133 in hypertension require investigations (46). Hence, conducting large-scale studies to determine the risk of hypertension based on *MTHFR* methylation and single nucleotide polymorphism in Taiwanese and other populations is worthwhile. Therefore, in the current, we evaluated the independent and interactive association of *MTHFR* rs1801133 single nucleotide polymorphism and *MTHFR* promoter methylation with hypertension among Taiwanese adults.

## Materials and methods

### Study participants

The Taiwan Biobank (TWB) collected the data used in the current study. The TWB project is a community-based study aimed at identifying disease biomarkers and underlying mechanisms through the integration of lifestyle, environmental, and genetic data (57, 58). This human biological database provides data for biomedical research in Taiwan (58). The TWB project enrolls only Taiwanese adults aged 30–70 years with no previous diagnosis of cancer (58). We initially included 1,241 individuals with methylation data in our study. However, we excluded 3 people from the final analysis because of missing data on exercise, BMI, and waist circumference. The final study sample included 1,238 participants, comprising 157 hypertensive and 1,081 non-hypertensive individuals. All participants signed informed consent forms before data collection. The Institutional Review Boards of Cathay General Hospital (CGH-P109032 and CGH-P10941) and Chung Shan Medical University Hospital (CS1–20009) approved this study.

### Genetic and epigenetic data

The Axiom Genome-Wide Array Plate chip system (Affymetrix Inc., Santa Clara, CA, USA) TWB (V2.0) chip was used for whole-genome genotyping. We used *MTHFR* rs1801133 (with genotypes CC, CT, and TT and minor allele T) in the current study because it is a well-established candidate for hypertension in several populations (10, 19, 47–52). The Hardy-Weinberg Equilibrium for *MTHFR* rs1801133 in the control group was 0.5999. The Illumina Infinium Methylation EPIC Bead Chip (Illumina Inc. San Diego, CA, USA) was used to determine whole blood-DNA methylation. We used *MTHFR* CpG sites at TSS1500 and TSS200 as promoter methylation data in our study (59, 60). Analysis of methylation data, including normalization, correction for batch effect, and cell-type heterogeneity was done using the ChAMP package.

### Definition of hypertension and covariates

We obtained information on physician-diagnosed hypertension, sex, age, cigarette smoking, alcohol intake, and exercise using self-reported responses to the TWB questionnaires.

Cigarette smokers were those who have ever smoked cigarettes continuously for at least six months. Alcohol drinkers included those individuals who reported a regular habit of drinking alcohol continuously for at least six months. Having a regular exercise habit meant engaging in physical activities (lasting more than thirty minutes) at least three times per week. We calculated the BMI using the standard formula: weight/height squared (kg/m^2^). Metabolic components were divided into normal and abnormal values based on the cutoffs used by the Taiwan Ministry of Health and Welfare and the American Heart Association (AHA)/National Heart, Lung, and Blood Institute (NHLBI) (61, 62). Waist circumferences <90 cm in men and <80 cm in women were considered normal, while values ≥90 cm in men and ≥80 cm in women were considered abnormal. Systolic blood pressure (SBP) <130 mmHg was normal, while ≥130 mmHg was abnormal. Diastolic blood pressure (DBP) <85 mmHg was normal, while ≥85 mmHg was abnormal. Fasting blood glucose (FBG) <100 mg/dl was normal, while ≥100 mg/dl was abnormal. Triglyceride <150 mg/dl was normal and ≥150 mg/dl was abnormal. High-density lipoprotein cholesterol levels ≥40 mg/dl in men and ≥50 mg/dl in women were normal, and <40 mg/dl in men and <50 mg/dl in women were abnormal.

### Statistical analyses

We evaluated the distribution of demographic data among individuals with the rs1801133 genotypes (CC, CT, and TT) using the student’s t-test (for continuous variables) and the chi-square test (for categorical variables). We used multiple logistic regression in determining the association of *MTHFR* rs1801133 and *MTHFR* promoter methylation quartiles (≥Q3 vs. Q2–Q3, Q1–Q2, and <Q1) with hypertension. We also used multiple logistic regression analysis to assess the interaction between *MTHFR* rs1801133 and *MTHFR* promoter methylation. We assessed the relationship between *MTHFR* rs1801133 and hypertension using the dominant (CC vs. CT + TT), recessive (CC + CT vs. TT), additive (CC/CT/TT), and log additive models (CC vs. CT and TT). The adjusted covariates were sex, age, body mass index, cigarette smoking, alcohol drinking, exercise, and metabolic components (waist circumference, systolic blood pressure, diastolic blood pressure, fasting blood glucose, triglycerides, and high-density lipoprotein cholesterol). SAS 9.4 software (SAS Institute, Cary, NC, USA) and PLINK 1.90 beta (Shaun Purcell & Christopher Chang, URL: www.cog-genomics.org/plink/1.9/) were used for data management and analyses. *P* < 0.05 was the threshold for statistical significance.

## Results

Table 1 illustrates the basic characteristics of the study participants stratified by the *MTHFR* rs1801133 genotypes (CC, CT, and TT). The proportion of individuals with hypertension was significantly different according to *MTHFR* rs1801133 genotypes (*P* < 0.0001). The *MTHFR* promoter methylation levels (means ± standard deviations) were also significantly different in terms of rs1801133 genotypes (*P* = 0.0065). The CC genotype had the lowest level (0.1375 ± 0.0064) followed by CT (0.1379 ± 0.0068) and TT (0.1395 ± 0.0063). The *MTHFR* promoter methylation quartiles were β < 0.1338 (<Q1), 0.1338 ≤ β < 0.1385 (Q1–Q2), 0.1385 ≤ β < 0.1423 (Q2–Q3), and β ≥ 0.1423 (≥Q3). The distribution (proportion) of individuals in the various quartiles was significantly different based on *MTHFR* rs1801133 genotypes (*P* = 0.0317).

**Table 1:** Demographic characteristics of the study participants according to the *MTHFR* rs1801133 genotypes.

Variable	rs1801133-CC (*n* = 622)	rs1801133-CT (*n* = 494)	rs1801133-TT (*n* = 122)	*P*
Hypertension				<0.0001
No	558 (89.71)	432 (87.45)	91 (74.59)	
Yes	64 (10.29)	62 (12.55)	31 (25.41)	
MTHFR promoter methylation level (β)	0.1375 ± 0.0064	0.1379 ± 0.0068	0.1395 ± 0.0063	0.0065
MTHFR promoter methylation quartiles				0.0317
≥Q3 (β ≥ 0.1422630)	135 (21.70)	134 (27.13)	41 (33.61)	
Q2–Q3 (0.1384618 ≤ β < 0.1422630)	155 (24.92)	118 (23.89)	35 (28.69)	
Q1–Q2 (0.1338376 ≤ β < 0.1384618)	170 (27.33)	117 (23.68)	24 (19.67)	
<Q1 (0 ≤ β < 0.1338376)	162 (26.05)	125 (25.30)	22 (18.03)	
Sex				0.1057
Women	313 (50.32)	248 (50.20)	49 (40.16)	
Men	309 (49.68)	246 (49.80)	73 (59.84)	
Age (years)	48.9614 ± 11.1913	49.2733 ± 11.3008	50.8525 ± 10.7954	0.2339
Body mass index (kg/m^2^)	24.2963 ± 3.7537	24.4043 ± 3.6990	25.0262 ± 3.9967	0.1461
Cigarette smoking				0.5989
Never	470 (75.56)	367 (74.29)	87 (71.31)	
Ever	152 (24.44)	127 (25.71)	35 (28.69)	
Alcohol drinking				0.6022
Never	559 (89.87)	449 (90.89)	113 (92.62)	
Ever	63 (10.13)	45 (9.11)	9 (7.38)	
Regular exercise				0.1820
No	371 (59.65)	282 (57.09)	62 (50.82)	
Yes	251 (40.35)	212 (42.91)	60 (49.18)	
Metabolic components				
Waist circumference (cm)				0.8030
Normal (men: <90, women: <80)	373 (59.97)	287 (58.10)	71 (58.20)	
Abnormal (men: ≥90, women: ≥80)	249 (40.03)	207 (41.90)	51 (4.80)	
Systolic blood pressure (mmHg)				0.0600
Normal (<130)	498 (80.06)	383 (77.53)	86 (70.49)	
Abnormal (≥130)	124 (19.94)	111 (22.47)	36 (29.51)	
Diastolic blood pressure (mmHg)				0.0486
Normal (<85)	542 (87.14)	409 (82.79)	98 (80.33)	
Abnormal (≥85)	80 (12.86)	85 (17.21)	24 (19.67)	
Fasting blood glucose (mg/dl)				0.0617
Normal (<100)	489 (78.62)	376 (76.11)	84 (68.85)	
Abnormal (≥100)	133 (21.38)	118 (23.89)	38 (31.15)	
Triglycerides (mg/dl)				0.5642
Normal (<150)	501 (80.55)	405 (81.98)	103 (84.43)	
Abnormal (≥150)	121 (19.45)	89 (18.02)	19 (15.57)	
High-density lipoprotein cholesterol (mg/dl)				0.1838
Normal (men: ≥40, women: ≥50)	465 (74.76)	366 (74.09)	100 (81.97)	
abnormal (men: <40, women: <50)	157 (25.24)	128 (25.91)	22 (18.03)	

Continuous variables are presented as means ± standard deviations and categorical variables as *n* (%).

*MTHFR*, methylenetetrahydrofolate reductase; β, beta-value for *MTHFR* promoter methylation.

Table 2 shows the risk of hypertension according to the *MTHFR* rs1801133 genotypes (CC vs. CT and TT) and *MTHFR* promoter methylation (≥Q3 vs. Q2–Q3, Q1–Q2, and <Q1). The TT genotype was significantly associated with a higher risk of hypertension (OR; 95% CI = 2.718; 1.503–4.914), while the CT genotype was not. The association of CT and TT with hypertension was dose-dependent (*P-trend *= 0.0041). All the *MTHFR* methylation quartiles were not significantly associated with hypertension. However, the interaction between the *MTHFR* promoter methylation quartiles and rs1801133 was significant (*P* = 0.0323). Older age, higher SBP, and higher FBG were significantly associated with a higher risk of hypertension: OR; 95% CI = 1.061; 1.032–1.092 for age, 5.524; 3.488–8.746 for SBP, and 2.235; 1.476–3.382 for FBG.

**Table 2:** Association of *MTHFR* rs1801133 SNP and *MTHFR* promoter methylation with hypertension.

	OR	95% CI	*P*
*MTHFR* rs1801133 (ref: CC)			
CT	1.177	0.765–1.809	0.4587
TT	2.718	1.503–4.914	0.0009
*P-trend*	0.0041		
*MTHFR* promoter methylation (ref: ≥Q3)			
Q2–Q3	1.222	0.692–2.159	0.4895
Q1–Q2	1.627	0.872–3.035	0.1263
<Q1	1.607	0.737–3.503	0.2329
Sex (ref: women)			
Men	1.285	0.546–3.025	0.5658
Age	1.061	1.032–1.092	<0.0001
Body mass index	1.051	0.978–1.129	0.1749
Cigarette smoking (ref: never)			
Ever	0.908	0.560–1.472	0.6965
Alcohol drinking (ref: never)			
Ever	1.121	0.611–2.058	0.7114
Regular exercise (ref: no)			
Yes	1.298	0.860–1.961	0.2146
Metabolic components			
Waist circumference (ref: normal)			
Abnormal	0.956	0.567–1.612	0.8646
Systolic blood pressure (ref: normal)			
Abnormal	5.524	3.488–8.746	<0.0001
Diastolic blood pressure (ref: normal)			
Abnormal	1.159	0.695–1.931	0.5720
Fasting blood glucose (ref: normal)			
Abnormal	2.235	1.476–3.382	0.0001
Triglycerides (ref: normal)			
Abnormal	1.518	0.928–2.484	0.0965
High-density lipoprotein cholesterol (ref: normal)			
Abnormal	1.356	0.840–2.190	0.2123
*MTHFR* rs1801133^a^*MTHFR* promoter methylation	*P* = 0.0323		

MTHFR, methylenetetrahydrofolate reductase; SNP, single nucleotide polymorphism; OR, odds ratio; CI, confidence interval; ref, reference.

^a^ interaction term.

Table 3 shows the association between *MTHFR* promoter methylation and hypertension stratified by the rs1801133 genotypes. Compared to the reference quartile (≥Q3), lower *MTHFR* promoter methylation quartiles were significantly associated with a higher risk of hypertension among individuals carrying the CC genotypes. The ORs (95% CIs) were 3.225 (1.140–9.124), 4.177 (1.424–12.247), and 8.645 (2.513–29.739) for Q2–Q3, Q1–Q2, and <Q1, respectively. The trend test was significant (*P-trend* = 0.0009).

**Table 3:** Association between *MTHFR* promoter methylation and hypertension stratified by *MTHFR* rs1801133 genotypes.

	rs1801133-CC (*n* = 622)			rs1801133-CT (*n* = 494)			rs1801133-TT (*n* = 122)		
	OR	95% CI	*P*	OR	95% CI	*P*	OR	95% CI	*P*
*MTHFR* promoter methylation (ref: ≥Q3)									
Q2–Q3	3.225	1.140–9.124	0.0273	0.553	0.210–1.454	0.2297	1.570	0.258–9.550	0.6243
Q1–Q2	4.177	1.424–12.247	0.0092	0.777	0.287–2.108	0.6208	1.870	0.162–21.634	0.6164
<Q1	8.645	2.513–29.739	0.0006	0.297	0.075–1.186	0.0857	0.376	0.022–6.540	0.5017
*P-trend*	0.0009			–			–		
Sex (ref: women)									
Men	0.536	0.135–2.132	0.3757	2.231	0.566–8.800	0.2518	1.064	0.042–27.072	0.9698
Age	1.081	1.035–1.130	0.0005	1.025	0.977–1.075	0.3105	1.133	1.008–1.273	0.0367
Body mass index	1.082	0.963–1.215	0.1853	0.977	0.869–1.099	0.6984	1.057	0.828–1.348	0.6583
Cigarette smoking (ref: never)									
Ever	0.757	0.352–1.628	0.4754	0.818	0.368–1.816	0.6212	2.312	0.423–12.641	0.3336
Alcohol drinking (ref: never)									
Ever	0.811	0.308–2.130	0.6702	2.724	1.018–7.292	0.0460	2.662	0.218–32.475	0.4429
Regular exercise (ref: no)									
Yes	1.292	0.685–2.435	0.4290	1.465	0.717–2.993	0.2949	1.268	0.275–5.853	0.7613
Metabolic components									
Waist circumference (ref: normal)									
Abnormal	0.810	0.355–1.851	0.6179	1.420	0.613–3.292	0.4135	0.751	0.112–5.046	0.7682
Systolic blood pressure (ref: normal)									
Abnormal	5.284	2.528–11.043	<0.0001	10.779	4.887–23.773	<0.0001	5.988	1.428–25.114	0.0144
Diastolic blood pressure (ref: normal)									
Abnormal	1.060	0.454–2.472	0.8936	1.274	0.570–2.848	0.5553	0.644	0.132–3.151	0.5873
Fasting blood glucose (ref: normal)									
Abnormal	2.527	1.321–4.835	0.0051	1.696	0.821–3.504	0.1536	3.401	0.934–12.384	0.0634
Triglycerides (ref: normal)									
Abnormal	1.492	0.666–3.340	0.3307	1.769	0.768–4.072	0.1801	1.517	0.311–7.394	0.6059
High-density lipoprotein cholesterol (ref: normal)									
Abnormal	1.203	0.565–2.561	0.6313	1.282	0.567–2.895	0.5507	1.883	0.324–10.951	0.4813

*MTHFR*, methylenetetrahydrofolate reductase; OR, odds ratio; CI, confidence interval, ref: reference.

The Supplementary Material shows the association between *MTHFR* rs1801133 and hypertension based on the dominant, recessive, and additive models. Using the dominant model (CC vs. CT + TT), the risk of hypertension was not significant (Supplementary Table S1-1). Nonetheless, the interaction between the *MTHFR* rs1801133 and promoter methylation was significant. Stratification by the genotypes revealed a significant association between hypertension and lower *MTHFR* promoter methylation quartiles among individuals carrying the CC genotypes (Supplementary Table S1-2). Using the recessive model (CC + CT vs. TT), the risk of hypertension was significantly higher among individuals carrying the TT genotype: OR; 95% CI = 2.513; 1.446–4.368 (Supplementary Table S2-1). The association between hypertension and *MTHFR* rs1801133 based on the additive model was also significant: OR; 95% CI = 1.519; 1.142–2.022 (Supplementary Table S3-1).

## Discussion

Improving hypertension prevention, diagnosis, and treatment requires pinpointing the associated genetic variants involved in the disease’s susceptibility, progression, and severity (38). As an integrated functional genomics approach, epigenetics could delineate some molecular mechanisms behind human diseases (63). However, knowledge about genetic and epigenetic events involved in disease susceptibility is scarce (36). In the current study, the risk of hypertension was significantly higher among individuals with the TT genotype compared to the CC genotype. Moreover, lower *MTHFR* promoter methylation levels were dose-dependently associated with a higher risk of hypertension among carriers of the CC genotype. To our knowledge, this is the first study to suggest the possible role of the *MTHFR* rs1801133 variant and promoter methylation in the pathogenesis of hypertension among Taiwanese. These findings add insights into some of the genetic and epigenetic mechanisms behind the disease onset.

The *MTHFR* rs1801133 variant interferes with the folate metabolic pathway by reducing the activity and thermostability of the *MTHFR* enzyme, thereby disrupting the methylation process (64). The interference in folate metabolism is associated with higher plasma homocysteine (Hcy) or hyperhomocysteinemia (65–67). Hyperhomocysteinemia increases oxidative stress and disrupts the elasticity of the vascular wall, causing endothelial damage, hypertension, and other complications (68–71).

The significantly positive association between the *MTHFR* rs1801133 TT genotype and hypertension observed in the current study is concurrent with previous findings. For instance, several meta-analyses involving individuals of different ethnic backgrounds found the TT genotype and the T allele of *MTHFR* rs1801133 as hypertension-susceptible variants (45, 72–74). A meta-analysis found a positive relationship between *MTHFR C677T* and hypertension but concluded that the findings were not robust enough (75). Another meta-analysis found a significantly positive relationship between the SNP and hypertension among Caucasians and East Asians (76). Several population-based studies also confirmed the positive relationship between the TT genotype or the T allele with hypertension. For instance, the T allele was associated with a higher risk of hypertension in Taiwanese (48), Chinese (47, 50, 77), Indians (78), and Australian Caucasians (52), and the TT genotype in Moroccans (51). Nonetheless, the variant was not significantly associated with hypertension in Algerians (79), South Africans (80), Chinese (54, 81), Danish (53), Caucasians (55), Japanese (56), Black Africans (76), Latinos (76), Sri Lankans (76), and Indians (76). We believe that the conflicting results between our study and those previously reported could, in part, be due to differences in ethnicities, sample sizes, and study designs.

The mechanism behind the link between *MTHFR* rs1801133 and hypertension is unclear. Epigenetic changes in the regulatory region, especially *MTHFR* promoter methylation levels are believed to affect gene expression, regardless of genetic mutations (82). Moreover, DNA methylation and epigenetic pathways are reversible and believed to be potential preventive and therapeutic targets in hypertension management (15). DNA methylation is a suggested epigenetic and pathological mechanism underpinning the role of genetic variants in disease susceptibility and progression (83). Hypomethylation related to folic acid was associated with the CC genotype of rs1801133, suggesting the possible epigenetic role of *MTHFR* (84). Moreover, the promoter activity of the regulatory sequence of *MTHFR* caused by rs1801133 was drastically reduced by *in vitro* methylation (21). Since *MTHFR* promotor methylation regulates *MTHFR* expression, it could be one of the mechanisms behind the effect of *MTHFR* variants on hypertension (21, 22). A trans-ancestry study found significant relationships between *MTHFR* and DNA methylation. The authors suggested that DNA methylation may be involved in the regulatory pathway connecting common genetic variants with blood pressure and multiple phenotypes (85).

In the current study, lower *MTHFR* promoter methylation levels were dose-dependently associated with a higher risk of hypertension among carriers of the CC genotype. So far, no studies have studied the risk of hypertension in relation to both *MTHFR* promoter methylation and *MTHFR* rs1801133. Our results support the view that DNA methylation might be involved in the association between the *MTHFR* gene and hypertension. *MTHFR* hypomethylation was associated with lower levels of *MTHFR* and higher levels of homocysteine (82, 86). *MTHFR* promoter hypermethylation was protective against ischemic stroke, implying that it could be a diagnostic marker for the disease (86). It is worth noting that hypertension is a risk factor for ischemic stroke (87). A randomized controlled trial (RCT) found riboflavin, a cofactor for *MTHFR*, as a modulator of global and gene-specific methylation in adults carrying the *MTHFR* TT genotype (88). Another RCT found that riboflavin can alter the DNA methylation profiles of some hypertension-related genes in adults carrying the *MTHFR* 677TT genotype (89).

In our study, alcohol drinkers with the CT genotype had a higher risk of hypertension. Alcohol drinking is positively associated with hypertension (24, 90–93). However, this relationship might differ based on the quantity consumed and frequency of intake. For instance, in a systematic meta-analysis of cohort studies among Koreans, Japanese, and Americans, alcohol intake of 50–100 g per day was associated with a higher risk of hypertension in both men and women (24). Nonetheless, an intake of <5 g per day had a linear dose-dependent relationship with hypertension in men and a J-shaped relationship in women, suggesting that low alcohol intake might protect against hypertension in women (24). In another meta-analysis of cohort studies, men who consumed at least one alcoholic drink per day had a significantly higher risk of hypertension (94). However, women who consumed 1 or 2 drinks per day did not have a significant risk (94). Similar to our results, fasting blood glucose (90, 95, 96) and age (90, 96–99) were associated with a higher risk of hypertension. Even though age is a well-documented promoter of hypertension (99), its relationship with blood pressure (a marker of hypertension) is inconsistent (90). That is, the trends in the relationship of age with SBP and DBP vary (100–104). As an example, the Framingham Heart Study showed different trends in SBP and DBP between ages 30 and 84 (103). In a study, SBP continuously increased with age while DBP increased, peaked at 50, and decreased between 60 and 80 years (100).

A limitation of the current study is that our data source did not have data on homocysteine. As such, we did not include such data in our analysis.

## Conclusion

*MTHFR* rs1801133 TT was independently associated with a higher risk of hypertension. This suggests that the TT genotype might elevate the risk of hypertension, regardless of methylation. However, *MTHFR* promoter methylation was not significantly associated with hypertension. Nonetheless, its interaction with *MTHFR* rs1801133 was significant. Based on genotypes, lower promoter *MTHFR* levels were associated with a higher risk of hypertension among individuals with the CC genotype. Our findings suggest that *MTHFR* rs1801133 and *MTHFR* promoter methylation could jointly influence hypertension susceptibility. Therefore, integrating genetic and epigenetic markers of hypertension improves risk stratification, which could enhance the implementation of targeted management strategies.

## Data Availability

The data analyzed in this study is subject to the following licenses/restrictions: The data that support the findings of this study are available from Taiwan Biobank but restrictions apply to the availability of these data, which were used under license for the current study, and so are not publicly available. Data are however available from the corresponding author upon reasonable request and with permission of Taiwan Biobank. Requests to access these datasets should be directed to Y-PL, Liawyp@csmu.edu.tw.
