# Supplementary material for: Susceptibility to hypertension based on MTHFR rs1801133 single nucleotide polymorphism and MTHFR promoter methylation
Source: Front Cardiovasc Med. 2023 Oct 2;10:1159764. doi: 10.3389/fcvm.2023.1159764 (PMC10577234; doi:10.3389/fcvm.2023.1159764)
Supplement: Supplementary file 1 [file Datasheet1.pdf]

Supplementary Table S1-1. Association of MTHFR rs1801133 (dominant model) and MTHFR promoter methylation with hypertension.

	OR	95% CI	P-value
**MTHFR rs1801133** (ref: CC)			
CT+TT	1.439	0.967-2.141	0.0728
**MTHFR promoter methylation** (ref: ≥Q3)			
Q2-Q3	1.242	0.706-2.186	0.4516
Q1-Q2	1.582	0.851-2.942	0.1471
<Q1	1.584	0.730-3.436	0.2445
P-trend		0.1708	
**Sex** (ref: women)			
men	1.332	0.571-3.106	0.5075
**Age**	1.059	1.030-1.089	<0.0001
**Body mass index**	1.056	0.983-1.134	0.1378
**Cigarette smoking** (ref: never)			
ever	0.913	0.565-1.474	0.7086
**Alcohol drinking** (ref: never)			
ever	1.097	0.601-2.002	0.7628
**Exercise** (ref: no)			
yes	1.289	0.855-1.943	0.2248
**Metabolic components**			
waist circumference (ref: normal)			
abnormal	0.925	0.551-1.555	0.7693
systolic blood pressure (ref: normal)			
abnormal	5.617	3.556-8.870	<0.0001
diastolic blood pressure (ref: normal)			
abnormal	1.142	0.687-1.897	0.6084
fasting glucose (ref: normal)			
abnormal	2.248	1.489-3.395	0.0001
triglycerides (ref: normal)			
abnormal	1.480	0.907-2.415	0.1166
high-density lipoprotein cholesterol (ref: normal)			
abnormal	1.307	0.812-2.103	0.2702
MTHFR rs1801133×MTHFR promoter methylation		p-value = 0.0295	

MTHFR: methylenetetrahydrofolate reductase, OR: odds ratio, CI: confidence interval, ref: reference, *: interaction term.

Supplementary Table S1-2. Association between MTHFR promoter methylation and hypertension stratified by MTHFR rs1801133 genotypes.

	**rs1801133-CC (n = 622)**			**rs1801133-CT+TT (n = 616)**		
	**OR**	**95% CI**	**P-value**	**OR**	**95% CI**	**P-value**
**MTHFR promoter methylation** (ref: ≥Q3)						
Q2-Q3	3.225	1.140-9.124	0.0273	0.726	0.348-1.517	0.3946
Q1-Q2	4.177	1.424-12.247	0.0092	0.825	0.356-1.912	0.6536
<Q1	8.645	2.513-29.739	0.0006	0.423	0.138-1.295	0.1318
P-trend		0.0009			-	
**Sex** (ref: women)						
men	0.536	0.135-2.132	0.3757	1.888	0.623-5.717	0.2610
**Age**	1.081	1.035-1.130	0.0005	1.041	1.001-1.083	0.0438
**Body mass index**	1.082	0.963-1.215	0.1853	1.018	0.923-1.123	0.7200
**Cigarette smoking** (ref: never)						
ever	0.757	0.352-1.628	0.4754	1.034	0.539-1.985	0.9190
**Alcohol drinking** (ref: never)						
ever	0.811	0.308-2.130	0.6702	1.721	0.754-3.930	0.1976
**Exercise** (ref: no)						
yes	1.292	0.685-2.435	0.4290	1.388	0.777-2.480	0.2674
**Metabolic components**						
waist circumference (ref: normal)						
abnormal	0.810	0.355-1.851	0.6179	1.106	0.541-2.259	0.7820
systolic blood pressure (ref: normal)						
abnormal	5.284	2.528-11.043	<0.0001	7.548	4.032-14.132	<0.0001
diastolic blood pressure (ref: normal)						
abnormal	1.060	0.454-2.472	0.8936	1.177	0.603-2.297	0.6327
fasting glucose (ref: normal)						
abnormal	2.527	1.321-4.835	0.0051	2.058	1.162-3.647	0.0134
triglycerides (ref: normal)						
abnormal	1.492	0.666-3.340	0.3307	1.638	0.825-3.253	0.1589
high-density lipoprotein cholesterol (ref: normal)						
abnormal	1.203	0.565-2.561	0.6313	1.307	0.673-2.537	0.4296

Supplementary Table S2-1. Association of MTHFR rs1801133 (recessive model) and MTHFR promoter methylation with hypertension.

	OR	95% CI	P-value
**MTHFR rs1801133** (ref: CC+CT)			
TT	2.513	1.446-4.368	0.0011
**MTHFR promoter methylation** (ref: ≥Q3)			
Q2-Q3	1.202	0.682-2.120	0.5249
Q1-Q2	1.588	0.855-2.951	0.1432
<Q1	1.560	0.718-3.386	0.2611
**Sex** (ref: women)			
men	1.280	0.544-3.013	0.5724
**Age**	1.062	1.032-1.092	<0.0001
**Body mass index**	1.051	0.978-1.129	0.1734
**Cigarette smoking** (ref: never)			
ever	0.908	0.560-1.472	0.6953
**Alcohol drinking** (ref: never)			
ever	1.113	0.607-2.041	0.7301
**Exercise** (ref: no)			
yes	1.309	0.867-1.975	0.2004
**Metabolic components**			
waist circumference (ref: normal)			
abnormal	0.954	0.566-1.609	0.8600
systolic blood pressure (ref: normal)			
abnormal	5.493	3.472-8.691	<0.0001
diastolic blood pressure (ref: normal)			
abnormal	1.175	0.706-1.955	0.5356
fasting glucose (ref: normal)			
abnormal	2.251	1.488-3.405	0.0001
triglycerides (ref: normal)			
abnormal	1.505	0.920-2.463	0.1035
high-density lipoprotein cholesterol (ref: normal)			
abnormal	1.365	0.846-2.203	0.7338
MTHFR rs1801133×MTHFR promoter methylation		p-value = 0.3426	

MTHFR: methylenetetrahydrofolate reductase, OR: odds ratio, CI: confidence interval, ref: reference, *: interaction term.

Supplementary Table S3-1. Association of MTHFR rs1801133 (additive model) and MTHFR promoter methylation with hypertension.

	OR	95% CI	P-value
**MTHFR rs1801133** (CC/CT/TT)	1.519	1.142-2.022	0.0041
**MTHFR promoter methylation** (ref: ≥Q3)			
Q2-Q3	1.250	0.709-2.204	0.4401
Q1-Q2	1.647	0.883-3.072	0.1163
<Q1	1.645	0.757-3.577	0.2091
**Sex** (ref: women)			
men	1.310	0.559-3.070	0.5344
**Age**	1.060	1.030-1.090	<0.0001
**Body mass index**	1.053	0.980-1.131	0.1607
**Cigarette smoking** (ref: never)			
ever	0.911	0.563-1.474	0.7047
**Alcohol drinking** (ref: never)			
ever	1.120	0.611-2.051	0.7138
**Exercise** (ref: no)			
yes	1.285	0.851-1.939	0.2325
**Metabolic components**			
waist circumference (ref: normal)			
abnormal	0.945	0.561-1.592	0.8326
systolic blood pressure (ref: normal)			
abnormal	5.591	3.534-8.845	<0.0001
diastolic blood pressure (ref: normal)			
abnormal	1.135	0.682-1.888	0.6255
fasting glucose (ref: normal)			
abnormal	2.222	1.469-3.360	0.0002
triglycerides (ref: normal)			
abnormal	1.516	0.928-2.476	0.0966
high-density lipoprotein cholesterol (ref: normal)			
abnormal	1.324	0.822-2.134	0.2485

MTHFR: methylenetetrahydrofolate reductase, OR: odds ratio, CI: confidence interval, ref: reference.
